# Correlation of Molecular Markers in High Grade Gliomas with Response to Chemo-Radiation

**DOI:** 10.31557/APJCP.2020.21.3.755

**Published:** 2020-03

**Authors:** Rohini Khurana, Satyajeet Rath, Harikesh Bahadur Singh, Madhup Rastogi, Sambit Swarup Nanda, Abhishek Chauhan, Mohammad Kaif, Nuzhat Hussain

**Affiliations:** 1 Department of Radiation Oncology,; 2 Department of Radiodiagnosis,; 3 Department of Neurosurgery,; 4 Department of Pathology, Dr. Ram Manohar Lohia Institute of Medical Sciences, Lucknow, Uttar Pradesh, India.

**Keywords:** High grade glioma, glioblastoma, GBM, anaplastic glioma, radiotherapy, 1p/19q codeltion, IDH 1 mutation

## Abstract

**Background:**

The standard of care in high grade glioma (HGG) is maximal safe surgical resection followed by adjuvant radiotherapy (RT) with/without chemotherapy. For anaplastic gliomas, studies have shown use of procarbazine, lomustine, vincristine (PCV) improves overall survival (OS) and progression free survival (PFS). Currently, there is substantial evidence that molecular markers strongly predict prognosis and response to treatment.

**Methods:**

Between January 2016 to January 2018, 42 patients were accrued and followed up till April 2019. The primary end points were to correlate molecular markers with response to therapy in terms of OS and PFS in HGG. The secondary end point was to evaluate frequency of 1p/19q codeletion, *IDH 1* mutation, ATRX deletion and p53 in HGG patients.

**Results:**

The median age was 46 years (range 18-67) with M:F ratio 30:12. The frequency of *IDH1* mutation,1p/19q codeletion, *p53* mutation and *ATRX* mutation were 42.8%, 16.6%, 42.8% and 14.2% respectively. All the seven patients with 1p/19q codeletion had *IDH1* mutation. Median follow up was 22 months. The 20-months PFS for different mutations were as follows; IDH1-mutated vs wild type: 53.6% vs 29.8%; p-0.035, 1p/19q codeleted vs non-codeleted: 85.7% vs 62.3%; p-0.011, p53 wild type vs mutated 32.1% vs 35.6%; p-0.035 and ATRX lost vs retained: 55.6% vs 53.3%; p- 0.369. The 20-months OS for *IDH1* mutated vs wild type: 82.4% vs 30.6%; p-0.014, 1p/19q codeleted vs non-codeleted: 85.7% vs 65.8%; p-0.104, p53 wild-type vs mutated 45.5% vs 73.9%; p-0.036 and ATRX lost vs retained: 100% vs 60.3%; p-0.087.

**Conclusion:**

Codeletion of 1p/19q with *IDH1* mutation in HGG is associated with a significantly favourable PFS. However, larger studies with longer follow up are required to evaluate OS and PFS in all the molecular subgroups.

## Introduction

Primary malignant brain tumors account for 1.4% of new cancer diagnosis in the United States and 2.7% of the deaths are due to central nervous system (CNS) tumors (Siegel et al., 2016). Incidence of CNS tumors in India ranges from 5 to 10 per 100,000 population with an increasing trend and accounts for 2% of all malignancies (Nair et al.,2015; Yeole et al., 2008). Astrocytomas (38.7%) were the most common primary tumors with the majority being high grade gliomas (59.5%) (Jalali et al., 2008; Nair et al., 2015; Yeole et al., 2008). Glioblastoma multiforme (GBM) accounts for more than half of all primary brain tumours (Jha et al., 2011).The 2007 World Health Organisation (WHO) Classification of tumours of the CNS was based on histopathological analysis (Louis et al., 2007). In 2014, the Haarlem consensus conference under auspices of International society of Neuro-Pathology (ISNP) established guidelines to incorporate molecular findings into histology of brain tumour diagnosis (Louis et al., 2014). Following it, the new CNS WHO classification came into picture in 2016 with major revisions based on molecular parameters to establish brain tumor diagnosis to illustrate prognostic behavior (Louis et al., 2016). However, there is no marked change in current standard of care in high grade gliomas (HGG). Maximal safe surgical resection (MSR) followed by radiotherapy (RT) with or without chemotherapy (CT) remains the standard of care. CT for anaplastic glioma (Anaplastic astrocytoma{AA}, Anaplastic oligodendroglioma {AO} and mixed Anaplastic Oligo-astrocytoma {AOA}) is not standardized even though use of procarbazine, lomustine, vincristine (PCV) improves overall survival (OS) and progression free survival (PFS) (Carincross et al., 2013; Van den bent et al., 2013). However, considering the OS and PFS improvement in GBM with the use of Temozolamide (TMZ) (Stupp et al., 2005), studies are being performed attempting the use of TMZ in Grade 2 and 3 gliomas (van den Bent et al., 2017). In this study we have tried to report the incidence of 1p/19q codeletion, *IDH 1* mutation, ATRX deletion and *p53* in HGGs in the Indian population and analyse variation of outcomes with respect to individual molecular marker and sub-group thereof. The rationale of the study was to predict response to standard chemo-radiation based on molecular markers in HGGs.

## Materials and Methods

This is a tertiary-hospital based prospective interventional study in which patients were accrued between January 2016 to January 2018 and followed up till April 2019.The project was approved by institutional ethics committee. The inclusion criteria in the current study were patients with histopathologically proven HGG, age ranging from 18-70 years, normal renal and liver function test and adequate bone marrow reserves. On the other hand, patients with prior history of any malignancy, previous administration of any form of chemotherapy or radiotherapy and patients unfit for chemotherapy and or radiotherapy were excluded from the study. Informed written consent was obtained from all patients. All the patients were assessed for symptoms and the duration of the presenting symptoms were recorded at the time of registration. Detailed neurological examination was performed. The Karnofsky performance score (KPS) were documented for all patients. All the patients were assessed radiologically using Gadolinium enhanced magnetic resonance imaging (MRI) to assess pre-treatment site, size of tumour and other features like edema, necrosis, calcification and vascularity. Histopathological evaluation for confirmation of diagnosis was done in all patients at the central pathology of the institute. The assessment also included molecular studies. Chromosome 1p and 19q deletion status were done by in Fluorescence situ hybridisation studies. IDH 1 mutation was analysed by immunohistochemistry (IHC) using mutation specific antibody. IHC was also used for assessing ATRX. All the patients underwent MSR followed by treatment with concurrent chemo-radiation (CRT) with standard dose and fractionation irrespective of O6-methylguanine-DNA methyl-transferase (MGMT) promotor methylation status followed by adjuvant chemotherapy. 

Total dose of RT was 60 Gray in 30 fractions (2 Gray per fraction, 5 days per week for total 6 weeks) with 3-dimensional conformal radiotherapy (3D-CRT) (LINAC, Elekta) along with concurrent TMZ followed by adjuvant TMZ for 6 months. The dose of TMZ was 75 mg/ m^2^ concurrent with RT on all days including public holidays followed by 6 cycles of adjuvant TMZ at 150 -200 mg/ m^2^ given 5 days every 28 days (Stupp et al., 2005). The patients were followed up monthly for 6 months, then bi-monthly for 6 months followed by three-monthly visits thereafter. They were assessed clinically on monthly basis during each follow-up. The post-treatment gadolinium enhanced MRI was done at 12 weeks after CRT completion. On the basis of response to treatment, cases were divided into complete responders (CR), partial responder (PR), stable disease (SD) and progressive disease (PD) as per the revised response assessment in neuro-oncology (RANO) criteria (Wen et al., 2010). The response assessment was done in the joint clinic with the opinion of neuro-surgeon, oncologist and radiologist. Patient data and treatment files were updated on each subsequent follow-up post-treatment. There were no loss to follow-up. The primary end point was to correlate relationship of various molecular markers 1p/19q codeletion, IDH 1 mutation, ATRX loss and *p53* mutation as well different subgroup clusters with similar marker expressions in terms of PFS and OS. The secondary end point was to evaluate frequency of 1p/19q codeletion, *IDH 1* mutation, ATRX deletion and *p53* in patients with HGGs. The hypothesis was that outcomes of HGGs treated with standard chemo-radiation may be different in terms of different molecular markers assessed and subgroups thereof, i.e., 1p/19q codeletion,* IDH 1* mutation, ATRX loss and p53 mutation. 

*Statistical analysis*

On the basis of response to treatment cases were be divided into CR, PR, SD and PD. The relationship of various molecular markers as well different subgroup clusters with similar marker expression were compared in terms of response to treatment and overall survival. Statistical analysis was performed using statistical package for sciences (SPSS version 23.0). PFS was defined as the time from the day of registration to date of progression or death. OS was measured from the date of registration to the date of death from any cause. Survival analysis was done by Kaplan Meier method with log rank test and all events were calculated from the date of registration.

## Results

Forty-two cases of HGG underwent four molecular studies. All the patients received adjuvant RT dose of 60 Gray with concurrent and adjuvant TMZ for six cycles. The patient characteristics are detailed in Table 1. The median age (range) was 46.5 years (18-67years); 30 (71.4%) were male. The presenting features included headache (95%), vomiting (65%) and seizures (48%). The median duration (mean, SD, range) of symptoms was 6 months (31, 19.8, 1-72). The median (mean, SD, range) diameter of disease was 5.0 cm (3.1, 3.2, 3.4-5.8) on gadolinium enhanced MRI. The median time (mean, SD, range) from the most recent surgery to start of radiotherapy being 1.5 months (2.8, 7.8, 5-6). Following immobilization patients were treated on a 6 /10 MV linear accelerator. The median RT dose (range) was 60Gy (59.4-60 Gray) given in 1.8-2.0 Gray fractions, 5 fractions/week for a duration of 6 weeks. The molecular profiling details are mentioned in Table 2.

**Figure 1:**
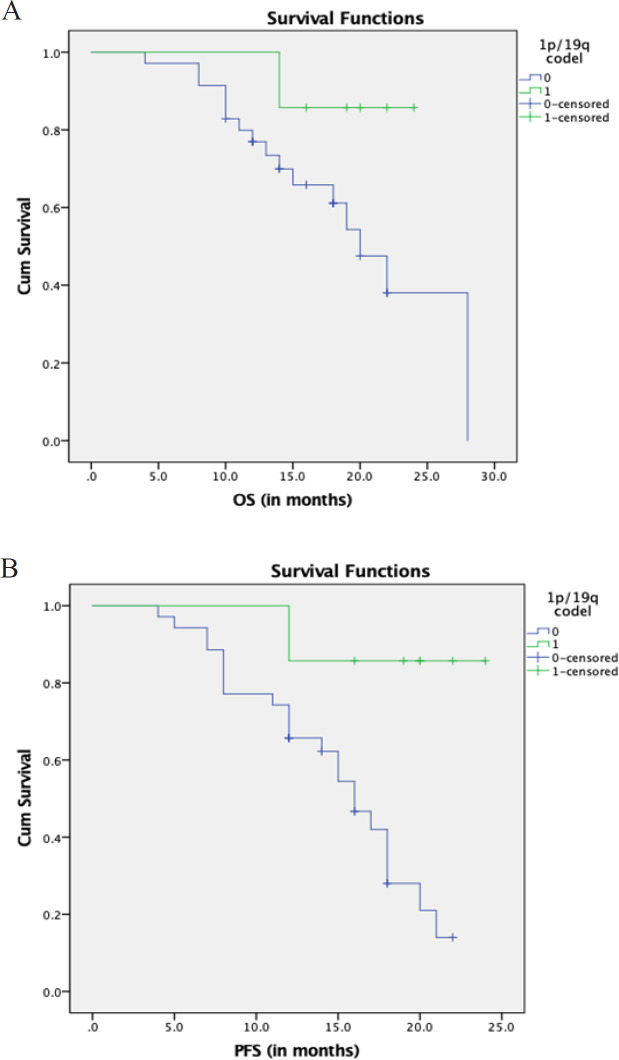
Figure Showing the Kaplan Meyer Curves for OS (A) and PFS (B) Variation with 1p/19q Co-deletion Status (codeleted vs noncodeleted)

Response assessment was performed as per RANO criteria (Wen et al., 2010) at a median follow up duration of 22 months was as follows: CR, PR and SD (n=18), PD (n=24). Of the 7 patients with 1p/19q codeletion, one patient had progressive disease at 1 year. All the seven patients with 1p/19q codeletion had *IDH1* mutation. Kaplan Meyer curves for the OS and PFS for all patients and 1p/19q codeleted subgroup is as shown in Figure 1 (A and B). Although there was no statistical difference in the OS (p-0.10), there was significant PFS improvement (85.7% vs 62.3%, p-0.01) with 1p/19q codeletion. The details of the variation in OS and PFS with all the molecular analysis are given in Table 3. The supplementary Table shows the variation in OS and PFS with various patient variables: age, gender, KPS, tumour size and tumour location.

## Discussion

MSR followed by adjuvant chemo-radiation is the most acceptable treatment strategy in the management of HGGs. EORTC 26899/NCIC established the role of concurrent and adjvuant TMZ as standard of care for glioblastoma (Stupp et al., 2005; Stupp et al., 2009). They reported increase in median survival to 14.6 months with CRT as compared to 12.1 months with only RT and 5 year OS increase from 1.9% to 9.8%. At the same time another study (Hegi et al., 2005) reported on subset analysis of 206 glioblastoma patients regarding epigenetic silencing of MGMT by methylation. Regardless of TMZ use, MGMT methylation was associated with improved OS (Median survival 15.3 vs 11.8 months). So, MGMT methylation is both prognostic and predictive for response to TMZ. Use of TMZ in unmethylated patients is controversial while some argued the subset was underpowered and patients may still benefit. Two landmark studies (RTOG 9402, EORTC 26951) were published regarding role of chemotherapy in anaplastic glioma in 2013. Cairncross (Cairncross et al., 2006; Cairncross et al., 2013; Cairncross et al., 2014) reported IDH mutated subgroup with 1p/19q codeleted median survival (MS) was 14.7 years as compared to 1p/19q intact (MS 5.5 years) and IDH wild type (MS-1.8 years) with use of chemotherapy (PCV), while in this trial chemotherapy was used prior to radiotherapy. Simultaneously EORTC 26951 (van den Bent et al., 2006; van den Bent et al., 2013) used adjuvant PCV after radiotherapy and compared with RT alone. They concluded that OS significantly improved with PCV (42.3 months vs 30.6 months). Significant improvement in PFS was noted in both 1p19q co-deleted (157 months vs 50 months) and 1p/19q non-codeleted (15 vs 9 months). IDH mutation and 1p/19q codeletions were independently significant on multivariate prognostic model. In our study, all the 1p/19q codeleted patients had *IDH1* mutations. We have observed an improved PFS (85.7% vs 62.3%, p-0.011) and OS (85.7% vs 65.8%, p–0.104) in *IDH* mutated 1p/19q codeleted subgroup as compared to those with 1p/19q non-codeleted. 

The role of TMZ in anaplastic gliomas has not been established. Of late, PCV has been replaced by TMZ in many studies performed in diffuse gliomas. A single arm phase II study, RTOG BR0131 (Vogelbaum MA et al., 2015), compared pre-RT and concurrent TMZ with RT with the historical cohort of RTOG 9402. Updates of this trial demonstrated 83% 6-year OS for co-deleted patients (compared with 67% for RTOG 9402, although no statistical difference was found). Similarly, the NOA-O4 study (Wick et al., 2016), which was investigating the optimum treatment sequence for anaplastic gliomas compared TMZ with PCV. In the subgroup analysis, they did not find significant difference in the OS between TMZ and PCV arms in co-deleted patients although there was a trend towards longer time to treatment failure for PCV. Also, the initial interim results of the CATNON study (van den Bent et al., 2017) show adjuvant TMZ was associated with improved survival (5-year OS: 55.9% vs 44.1%) in non-co-deleted anaplastic gliomas. The results have been further rebuffed in the second interim analysis (van den Bent et al., 2019). It shows adjuvant TMZ improves OS and CRT with concurrent TMZ shows trend towards improved OS in IDH mutated tumour. The results from four important trials of radiotherapy and chemotherapy have been discussed by Soltys et al. along with molecular grading of gliomas (Soltys et al., 2018). The future outcomes of studies like CODEL NCCTG N0577 (Jaeckle K et al., 2016) and detailed analysis of CATNON will further clarify the role of TMZ in HGG. RTOG 0131, NOA-04 and early result of CATNON all suggest this is a reasonable substitution to PCV. In the light of the above trials, we have tried to analyse the outcome in HGGs using RT with concurrent TMZ and correlate them with the various molecular markers.

Till now limited studies have been conducted in Indian patients regarding frequency of 1p/19q codeletion, IDH 1 mutation, ATRX deletion and *TP53* and further to correlate individual markers signature with response to chemoradiation and impact on overall survival in high grade gliomas. One study Jha et al., (2011) reported molecular profiling of GBMs in the Indian population. They reported that EGFR (37.3%) and PTEN (54.9%) mutations are relatively common in primary GBM, while *TP53* (66.7%) and IDH1 (44.4%) mutations were more common in secondary GBMs. *IDH 1* mutation were reported in 68.8%, 85.7% and 12.8% in grade 2, 3 and 4 gliomas, respectively. *IDH 1* mutation is present in over 80% of secondary GBMs (Yan et al., 2009), while *IDH2* gene have been reported in around 3% of gliomas (Hartmann et al., 2009). Another study (Parsons et al., 2008) has found improved survival with IDH1 mutations. The current study found* IDH1* mutations in 42.8% patients. The cases with IDH 1 mutations had better 20-month OS and 20-month PFS, 82.4% vs 30.6% (p-0.014) and 53.6% vs 29.8% (p-0.035), respectively. 

The incidence of 1p/19q co-deletions among GBMs is low compared to anaplastic oligodendrogliomas. We found 1p/19q codeletion in roughly 17% patients. 1p/19q co-deletions alone did not seem to be associated with good outcomes in one report with GBM (Kaneshiro et al., 2009, Smith et al., 2000), although another study reported GBM long-term survivors with 1p/19q co-deletions (Burton et al., 2002). The current study found non-significant improvement in 20-month OS (85.7% vs 65.8%, p-0.104), but significantly improved 20-month PFS (85.7% vs 62.3%, p-0.011) in 1p/1pq codeleted patients. In light of the fact that 1p/19q codeletion entails improved prognosis and chemosensitivity in anaplastic gliomas, and histo-pathological analysis alone is not sufficient for diagnosis anymore, genetic and molecular testing should be routinely performed for these tumours (Idbaih et al., 2007). The incidence of somatic p53 alterations in GBMs is around 85%, as reported in a study by the Cancer Genome Atlas (Cancer genome atlas, 2008). The present study found *p53* mutation in approximately 43% cases.

Summarising the data in the aforementioned literature, the role of molecular markers in the treatment of HGGs is still an intriguing matter (Clark et al., 2013). With the changing landscape of diagnosis of gliomas, the newer data is largely revolving around next generation sequencing (Parsons et al., 2008) and the Cancer genome atlas (Cancer genome atlas, 2008). Shortcomings of the current study include small sample size which did not allow assessment of various molecular subgroups possible in gliomas (Suzuki et al., 2015). MGMT methylation analysis was not available for all the patients. The optimal duration of treatment with adjuvant temozolamide, 6 months versus 12 months, is still not established. Tumour located in midline were not assessed separately (Weller et al., 2017). 

In conclusion, the current study has reported the incidence of various molecular markers in HGG and the outcomes after chemo-radiation with regards to these markers. With the integrated classification of diffuse gliomas as advocated by WHO (2016), this study was initiated to standardise treatment related decisions in HGGs. Codeletion of 1p/19q in HGG with IDH1 mutation is associated with a significantly favourable progression free survival (p=0.011). However, longer follow up with a larger cohort is required to assess overall survival (p=0.104). Studies with a larger number of patients and a longer follow up will help to answer the question.

**Table 1:** Patient Demographic Characteristics

Patient demographics	Values
Median age (Range) (in years)	46.5 years (18-67years)
Gender (Male: Female)	30:12:00
KPS (<70: 70 : >70)	04:34:04
Median duration of symptoms	9 months (1-72 months)
Location of disease (F: P: T)	19:13:10
Median diameter of disease	5.0 cm (3.4-5.8 cm)
Grade 3: Grade 4	23:19
AO: AA: AOA	12:04:07
Median time from Surgery to RT start	1.5 months (1-6)
Median RT dose range	60Gy (59.4-60 Gray)
CR+SD: PD	10:24

KPS, Karnofsky Performance Score; CR, Complete response; SD, Stable disease; PD, Progressive disease

**Table 2.** Frequency of Molecular Markers

Molecular Marker	n (%)
IDH1 Mutated	18/42 (42.8%)
1p/19q Codeletion	7/42 (16.6%)
p53 Mutated	18/42 (42.8%)
ATRX Loss	6/42 (14.2%)
IDH1mutated – 1p/19q Codeleted* (N=18)	7/18 (38.8%)

**Table 3:** Variation of OS and PFS with Various Molecular Markers

Molecular Marker	*OS	P value	*PFS	P value
IDH1	82.4% vs 30.6%	0.014	53.6% vs 29.8%	0.035
(Mutated vs Nonmutated)				
1p/19q Codeletion	85.7% vs 65.8%	0.104	85.7% vs 62.3%	0.011
(Codeleted vs Noncodeleted)				
p53	73.9% vs 45.5%	0.036	35.6% vs 32.1%	0.269
(Mutated vs Wild-type)				
ATRX	100% vs 60.3%	0.087	55.6% vs 53.3%	0.369
(Loss vs No loss)				

*The OS and PFS mentioned here are 20-month OS and 20-month PFS.

## Approval

The study was approved by Institutional ethics committee as an intramural project.
